# Editorial: Pain management in abdominal surgery

**DOI:** 10.3389/fsurg.2023.1175543

**Published:** 2023-03-20

**Authors:** Stefano Pontone, Marco Lauriola

**Affiliations:** ^1^ Department of Surgical Sciences, Faculty of Medicine and Dentistry, Sapienza University of Rome, Rome, Italy; ^2^ Department of Social and Developmental Psychology, “Sapienza” University of Rome, Rome, Italy

**Keywords:** pain, surgery, opioid, analgesia, laparoscopy, anestesia, lidocaine, TAP block

**Editorial on the Research Topic** Pain management in abdominal surgery

Postoperative pain is a common issue that patients face after abdominal surgery. Traditionally, opioids have been the basis of postoperative pain management (1). However, with increasing awareness of the risks associated with opioid use, such as addiction (2) and respiratory depression (3), alternative pain management strategies have been explored (Patil et al.) (4, 5). In recent years, the focus has shifted towards personalized, multimodal pain management strategies that combine pharmacological and non-pharmacological methods to provide optimal pain relief while reducing the side effects (6, 7). This Special Issue in Frontiers in Surgery brings together four randomized controlled trials (Yan et al. Yeh et al. Jemal et al. Ma et al.), two meta-analysis papers (Wang et al. Chen et al.), and one mini-review (Patil et al.) offering valuable insights into the efficacy and safety of analgesic techniques in abdominal surgery.

Yun Yan et al. emphasized the value of multimodal analgesia in lowering postoperative pain and opioid use following lumbar spine surgery. The trial assessed the effectiveness of an opioid-sparing multimodal method that included intravenous acetaminophen, gabapentin, and dexamethasone. The study concluded that multimodal analgesia should be considered as the first-line analgesic approach for postoperative pain in lumbar spine surgery based on the findings that this combination was successful in lowering postoperative pain and opioid intake.

Kuang-Yi Chang (Yeh et al.) and the meta-analysis by Weihua Wang et al. studied the efficiency of Transversus Abdominis Plane (TAP) block in lowering postoperative pain and enhancing recovery after hepatic cancer surgery and laparoscopic cholecystectomy, respectively. A local anesthetic injected into the fascial plane between the internal oblique and transversus abdominis muscles was used as part of the TAP block, a regional anesthesia method. The findings of both studies demonstrated that TAP block was effective in lowering postoperative pain levels, lowering usage of opioids, and speeding up recovery. These findings suggested that TAP block can be a useful method in multimodal analgesia strategies for postoperative pain control.

Bedru Jemal et al. showed that spinal morphine compared to TAP block provided greater lasting analgesia and patients needed fewer postoperative opioids. However, It is worth noting that using spinal morphine carries a higher risk of experiencing adverse effects like pruritus, nausea, and vomiting. Furthermore, Ping Wang (Ma et al.) provided evidence that TAP block was sufficient to enhance the analgesic effect and improve postoperative recovery time after laparoscopic radical resection of cervical cancer. This study highlighted the potential benefits of TAP block as a postoperative pain management option for patients undergoing laparoscopic radical resection of cervical cancer.

The Po-Chuan Chen et al. meta-analysis sheds light on the possible advantages of improving bowel function recovery following major colon surgery by utilizing Intravenous Infusion of Lidocaine (IVF-Lido). The results of the study imply that IVF-lido may have other advantages beyond its analgesic effects, including aiding bowel function recovery. The same study also proposed that IVF-lido may be a beneficial tool in reducing the amount of time required for recovery following major colon surgery.

In addition to the above-mentioned studies, which all looked into various pharmacological pain management techniques, Devpal Patil et al. reviewed three nonpharmacological approaches, such as cognitive-behavioral therapy, hypnosis, and relaxation techniques that have the potential for managing pain following surgery. Thus, to give patients more options and better results, non-pharmacological treatments should be considered as a complement to conventional pain management techniques. Future research should go precisely in the direction of testing the effectiveness of integrating psychological preparation interventions as an adjuvant treatment to achieve maximum postoperative pain control with minimal drug use.

Overall, all the studies included in the special issue offered new insights into the effectiveness and safety of various pain management techniques for abdominal surgical procedures. The results imply that tailored pain management strategies that consider patient traits and the analgesic being used may be more successful in helping patients control their postoperative pain. All studies also emphasized the importance of customized pain management, considering elements like patient characteristics and surgical techniques.

To maximize pain control and encourage recovery, analgesic medication should be customized to the needs of the patient. In addition, in specific patient populations, the use of ultrasound-guided TAP block (8), spinal morphine (9), and IVF-lido may be efficient choices for postoperative pain control (10). In line with previous research, these methods lessen the likelihood of opioid-related side effects such respiratory depression, nausea, and constipation by lowering the usage of opioids.

Before concluding it is worth remembering that the effectiveness of pain management might change based on patient characteristics, the nature of the surgery, and the type of pain. Also, technical factors, like the injection method, the volume and concentration of the local anesthetic utilized, and the number of injections given, might affect how pain management techniques may turn effective (11, 12). As a result, more research is needed to identify the best pain management strategy for various patient demographics following surgery.

In sum, the studies included in the special issue highlighted the need for more research to establish uniform protocols and confirm the efficacy of different analgesic approaches. The quality of patient care can be improved, and better postoperative outcomes can be achieved by a better understanding of postoperative pain management. Customized pain management techniques that consider patient characteristics, as well as the drug used, may be more effective in treating postoperative pain in patients. To maximize pain management and promote recovery, analgesic treatments should be customized to the unique demands of the patient and the procedure. Lastly, non-pharmacological techniques have shown promise in providing immediate relief from post-operative pain, and further research is needed to explore their potential benefits fully.
